# A Surveillance Mechanism Ensures Repair of DNA Lesions during Zygotic Reprogramming

**DOI:** 10.1016/j.cell.2016.11.009

**Published:** 2016-12-15

**Authors:** Sabrina Ladstätter, Kikuë Tachibana-Konwalski

**Affiliations:** 1 Institute of Molecular Biotechnology of the Austrian Academy of Sciences (IMBA), Vienna Biocenter, Dr. Bohr-Gasse 3, Vienna 1030, Austria

**Keywords:** zygote, reprogramming, checkpoint, cohesin, DNA damage repair

## Abstract

Sexual reproduction culminates in a totipotent zygote with the potential to produce a whole organism. Sperm chromatin reorganization and epigenetic reprogramming that alter DNA and histone modifications generate a totipotent embryo. Active DNA demethylation of the paternal genome has been proposed to involve base excision and DNA repair-based mechanisms. The nature and consequence of DNA lesions generated during reprogramming are not known. Using mouse genetics and chemical biology, we discovered that Tet3-dependent zygotic reprogramming generates paternal DNA lesions that are monitored by a surveillance mechanism. In vivo structure-function rescue assays revealed that cohesin-dependent repair of paternal DNA lesions prevents activation of a Chk1-dependent checkpoint that delays mitotic entry. Culturing conditions affect checkpoint stringency, which has implications for human in vitro fertilization. We propose the zygotic checkpoint senses DNA lesions generated during paternal DNA demethylation and ensures reprogrammed loci are repaired before mitosis to prevent chromosome fragmentation, embryo loss, and infertility.

## Highlights

• Active DNA demethylation during zygotic reprogramming produces paternal DNA lesions
• Paternal DNA lesions depend on Tet3 activity and require Scc1-cohesin for repair
• Inefficient repair of paternal DNA lesions activates a Chk1-mediated checkpoint
• A zygotic checkpoint coordinates repair with mitosis to protect genome stability

## Graphical Abstract

**Figure:**
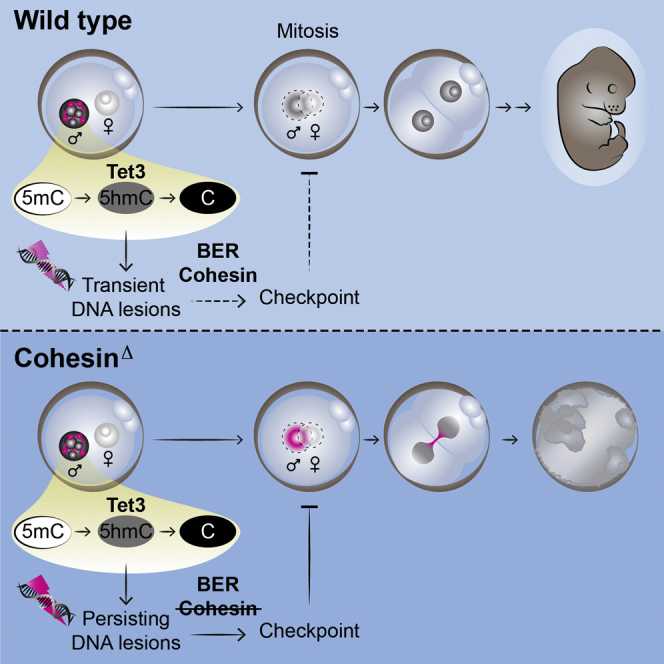

## Teaser

A surveillance mechanism that facilitates the repair of DNA lesions in the paternal genome during zygotic reprogramming has implications for in vitro fertilization.

## Introduction

Embryonic development begins with reprogramming to totipotency during the oocyte-to-zygote transition. Fusion of a terminally differentiated egg (metaphase II oocyte) and sperm elicits complex changes including chromatin remodeling and epigenetic reprogramming within the one-cell zygote. The most dramatic changes occur in the paternal genome, where compacted sperm chromatin is reorganized: protamines are exchanged for maternal nucleosomes shortly after fertilization (Rodman et al., 1981), distinct histone modifications are established (Burton and Torres-Padilla, 2010), and DNA is demethylated in G1 and S phases (Guo et al., 2014, Mayer et al., 2000, Oswald et al., 2000, Shen et al., 2014).

Sperm DNA is highly methylated at cytosines (5mC). Most sperm-derived 5mC is demethylated independently of DNA replication during the first zygotic cell cycle (Mayer et al., 2000, Oswald et al., 2000). The mechanism of active DNA demethylation utilized in zygotes is poorly understood. Active DNA demethylation can proceed through different repair-based mechanisms that generally involve modification of the 5mC, followed by excision of the modified base/nucleotide and replacement with cytosine (Seisenberger et al., 2013, Zhang and Zhu, 2012). During zygotic reprogramming, 5mC is modified to 5-hydroxymethylcytosine (5hmC) by the Tet3 hydroxylase (Gu et al., 2011, Guo et al., 2014, Inoue and Zhang, 2011, Shen et al., 2014, Wossidlo et al., 2011). This oxidized cytosine is either excised and replaced by unmodified cytosine, further modified, or passively diluted by DNA replication. Additionally, Tet3-independent mechanisms for 5mC loss exist (Amouroux et al., 2016).

How 5mC or its modified versions are actively replaced with cytosine in the zygote is unclear. It has been proposed that an unidentified DNA glycosylase removes modified cytosine and triggers recruitment of the base excision repair (BER) machinery (Cortellino et al., 2011, Guo et al., 2014, He et al., 2011, Santos et al., 2013). Consistent with an involvement of BER, chemical inhibition of the BER components Parp1 and APE1 affect paternal DNA demethylation (Hajkova et al., 2010). In addition, the essential BER component Xrcc1 is enriched on paternal chromatin (Hajkova et al., 2010), but whether it is required to repair DNA lesions generated by paternal DNA demethylation is not known. A repair-coupled pathway of active DNA demethylation would entail transient generation of DNA strand breaks. DNA double- and single-stranded breaks are marked by phosphorylated histone H2AX (γH2AX) (House et al., 2014), and γH2AX is widely used as surrogate marker for DNA lesions to overcome the technical challenge of detecting DNA breaks in single cells. Remarkably, γH2AX foci are detected at the time of paternal DNA demethylation (Wossidlo et al., 2010), suggesting that DNA breaks are indeed generated during zygotic reprogramming.

The involvement of other DNA repair pathways such as homologous recombination (HR) in addition to BER during zygotic reprogramming is hypothetical. Given that cohesin is a multifunctional complex with roles in higher-order chromatin structure, DNA damage repair and DNA damage-induced cell-cycle checkpoints (Hadjur et al., 2009, Kagey et al., 2010, Kim et al., 2002, Kitagawa et al., 2004, Seitan et al., 2011, Watrin and Peters, 2009, Wendt et al., 2008, Yazdi et al., 2002), it is conceivable that it might be especially important for zygotic development. Cohesin is known to be required for DNA repair by HR. HR requires the physical proximity of sister chromatids, which are held together by cohesin mediating sister chromatid cohesion (Losada et al., 1998, Uhlmann and Nasmyth, 1998). A role for cohesin in DNA repair beyond holding sister chromatids together has also been proposed, for example by stabilizing broken DNA ends or functioning as a recruitment platform (Jessberger, 2009). The precise role of cohesin during DNA repair independently of sister chromatid cohesion remains poorly understood. However, it has been shown that the Scc1 subunit of cohesin is sumoylated during the DNA damage response (Wu et al., 2012). This modification is not essential for cohesion but necessary for sister chromatid exchange (Wu et al., 2012), implying that a modified version of cohesin promotes DNA repair.

Whether the programmed DNA lesions generated during zygotic reprogramming signal to a cell-cycle checkpoint is not known. In principle, however, zygotes are capable of mounting a DNA damage response (DDR), at least in the presence of extensive DNA damage brought in by irradiated sperm (Gawecka et al., 2013, Pacchierotti et al., 2011). Given that zygotes harbor DDR mechanisms, we asked whether these might coordinate zygotic reprogramming with cell-cycle progression to ensure genome integrity in the single-cell embryo.

Here, we show that loss of the essential BER component Xrcc1 stabilized paternal DNA lesions generated during zygotic reprogramming. However, these DNA lesions were eventually repaired, suggesting that other pathways, such as HR or non-homologous end joining, may act redundantly with BER. We therefore considered whether cohesin, which is required for HR but not known to be involved in BER, would stabilize DNA lesions generated during reprogramming. Indeed, we find that DNA lesions detected in the paternal genome during DNA demethylation require cohesin for their repair. In addition, unrepaired lesions in zygotes lacking cohesin activate a Chk1-dependent checkpoint that delays entry into mitosis. Checkpoint activation is not solely a consequence of cohesin loss but depends specifically on the presence of the paternal genome and on Tet3-dependent DNA lesions generated during DNA demethylation. We propose that the zygotic checkpoint uncovered by cohesin loss senses DNA breaks generated by epigenetic reprogramming and links its completion with mitosis.

## Results

### Paternal DNA Lesions in G1 Phase Are Repaired by Base Excision Repair

**Figure 1:**
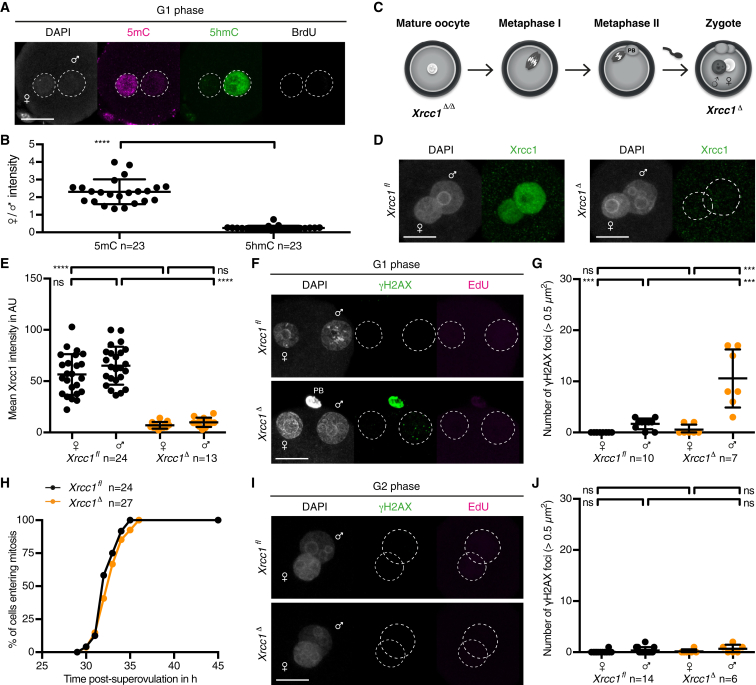
Paternal DNA Lesions Are Revealed by Lack of Xrcc1 during Active DNA Demethylation in G1 Phase Zygotes (A and B) Immunofluorescence analysis of global 5mC and 5hmC in wild-type G1 phase zygotes. (A) Representative images. (B) Quantification of maternal to paternal ratio of mean (B) 5mC and (C) 5hmC intensity. (C) *Xrcc1*^Δ^ zygotes are obtained after fertilization of *Xrcc1*^*Δ/Δ*^ oocytes with wild-type sperm. (D and E) Immunofluorescence detection of total Xrcc1 in G1 phase zygotes. (D) Representative images. (E) Quantification of mean Xrcc1 intensity in maternal and paternal pronuclei, respectively. (F and G) Analysis of γH2AX foci in G1 phase zygotes. (F) Representative images. (G) Quantification of γH2AX foci number in maternal and paternal pronuclei, respectively, in G1 phase. (H) Mitotic entry kinetics of zygotes scored according to nuclear envelope breakdown. (I and J) Immunofluorescence analysis of γH2AX foci in G2 phase zygotes that are fixed after 30 min EdU pulse to exclude cells that still undergo DNA replication. (I) Representative images. (J) Quantification of G2 phase γH2AX foci number in maternal and paternal pronuclei, respectively. Note for (A), (B), and (D)–(G), cells were cultured in continuous presence of BrdU or EdU from isolation until fixation to exclude cells that eventually started DNA replication. ^∗∗∗∗^p < 0.0001, ^∗∗∗^p < 0.001, ^ns^p > 0.5, calculated using unpaired t test (B and E) or Mann-Whitney test (G and J). All error bars indicate SD. AU, arbitrary units; PB, polar body. Scale bars, 20 μm. See also Figure S1.

Both the maternal and paternal genomes contain methylated cytosine at fertilization (Mayer et al., 2000, Oswald et al., 2000). To study mouse zygotic reprogramming and paternal DNA demethylation in the context of the cell cycle, we used the thymidine analogs 5-bromo-2’-deoxyuridine (BrdU) or 5-ethynyl-2′-deoxyuridine (EdU) to label DNA replication. We detected reduced levels of 5mC and increased levels of 5hmC in the paternal genome prior to S phase by immunofluorescent staining of zygotes (Figures 1A and 1B). Thus, active DNA demethylation of the paternal genome occurs in G1 phase, consistent with previous observations (Mayer et al., 2000, Oswald et al., 2000, Wossidlo et al., 2010).

**Figure S1:**
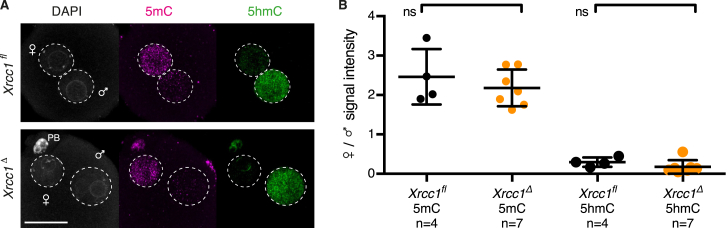
Immunofluorescence Analysis of 5mC and 5hmC Levels in *Xrcc1*^Δ^ Zygotes, Related to Figure 1 (A) Representative images of G2 phase zygotes. (B) Quantification of maternal to paternal ratio of mean 5mC and 5hmC intensity. ^ns^ p > 0.5, calculated using unpaired t test. n, number of cells. Scale bar, 20 μm.

To investigate whether active DNA demethylation entails a BER mechanism, we asked whether Xrcc1 is required for the repair of paternal DNA lesions in G1 phase zygotes. To achieve this, we deleted conditional (floxed) alleles of *Xrcc1* specifically in oocytes using *(Tg)Zp3*-Cre (Figure 1C). The *zona pellucida 3* (*Zp3*) promoter drives Cre recombinase expression during the 2–3 weeks of growth leading to a mature oocyte (Lan et al., 2004, Lewandoski et al., 1997). The first meiotic division occurs at ovulation and the second division is triggered by fertilization, producing a single-cell embryo or zygote. Because most proteins are provided by the oocyte and there is no essential transcription in the first cell cycle (Aoki et al., 1997, Hamatani et al., 2004), we designate the genotype of the zygote according to the maternal allele. To test whether Xrcc1 is required for DNA repair during the first cell cycle, we isolated zygotes from crosses of wild-type males with control *Xrcc1*^*fl/fl*^ or experimental *Xrcc1*^*fl/fl*^ *(Tg)Zp3-Cre* females. For simplicity, we refer to control *Xrcc1*^*fl(m)/+(p)*^ and knockout *Xrcc1*^*Δ(m)/+(p)*^ zygotes as *Xrcc1*^*fl*^ and *Xrcc1*^*Δ*^ zygotes, respectively. Xrcc1 was detectable in both nuclei of *Xrcc1*^*fl*^ zygotes whereas levels were decreased in *Xrcc1*^*Δ*^ zygotes (Figures 1D and 1E). Xrcc1 depletion had little or no effect on global DNA demethylation (Figure S1). To test whether Xrcc1 is required to repair paternal DNA lesions, we examined γH2AX foci in G1 phase zygotes. Few, if any, γH2AX foci are detected in maternal chromatin, suggesting that Xrcc1 depletion has little effect on chromatin integrity in oocytes (Figures 1F and 1G). Although few γH2AX foci are detected in paternal chromatin of *Xrcc1*^*fl*^ zygotes, presumably due to efficient repair, up to 10-fold more foci are detected on paternal chromatin of *Xrcc1*^*Δ*^ zygotes. Persistence of γH2AX foci in the absence of Xrcc1 provides the first functional evidence that paternal DNA lesions are repaired by BER and suggests that the lesions arise from base excision. We conclude that Xrcc1-mediated BER is necessary for repairing paternal DNA lesions in G1 phase zygotes.

To test whether paternal DNA lesions signal to the cell-cycle machinery, we performed time-lapse imaging of zygotes to determine kinetics of mitotic entry. *Xrcc1*^*fl*^ and *Xrcc1*^*Δ*^ zygotes entered mitosis with similar kinetics (Figure 1H), suggesting that no strong checkpoint has been elicited. There are several possible explanations, for instance paternal DNA lesions may not be sensed by a surveillance mechanism or may later be repaired. To distinguish between these possibilities, we asked whether paternal DNA lesions persist throughout interphase when Xrcc1 is depleted. We found that *Xrcc1*^*fl*^ and *Xrcc1*^*Δ*^ zygotes display little or no γH2AX foci in G2 phase (Figures 1I and 1J), suggesting that paternal DNA lesions are eventually repaired. This repair may be mediated by residual Xrcc1 in *Xrcc1*^Δ^ zygotes or by other DNA repair pathways that act redundantly later in the cell cycle. Overall, these data demonstrate a functional requirement for BER of paternal DNA lesions but their subsequent repair precludes an analysis of surveillance mechanisms monitoring zygotic reprogramming.

### Repair of Paternal DNA Lesions Requires Cohesin in Zygotes

To study how paternal DNA lesions generated during reprogramming are coordinated with the cell cycle, we asked whether their repair during interphase requires cohesin. Distinct cohesin complexes exist in eukaryotes, and a switchover of cohesin complexes occurs at the oocyte-to-zygote transition (Tachibana-Konwalski et al., 2010). In oocytes, cohesion is maintained by cohesin containing a heterodimer of Smc1/3 bridged by the meiosis-specific α-kleisin Rec8. After fertilization, cohesion is established and maintained by Scc1-containing cohesin in zygotes and somatic cells. Therefore, we considered it possible to produce *Scc1* knockout oocytes without perturbing meiotic chromosome segregation. Fertilization would produce Scc1-depleted zygotes for analysis of DNA lesions and cell-cycle progression.

**Figure S2:**
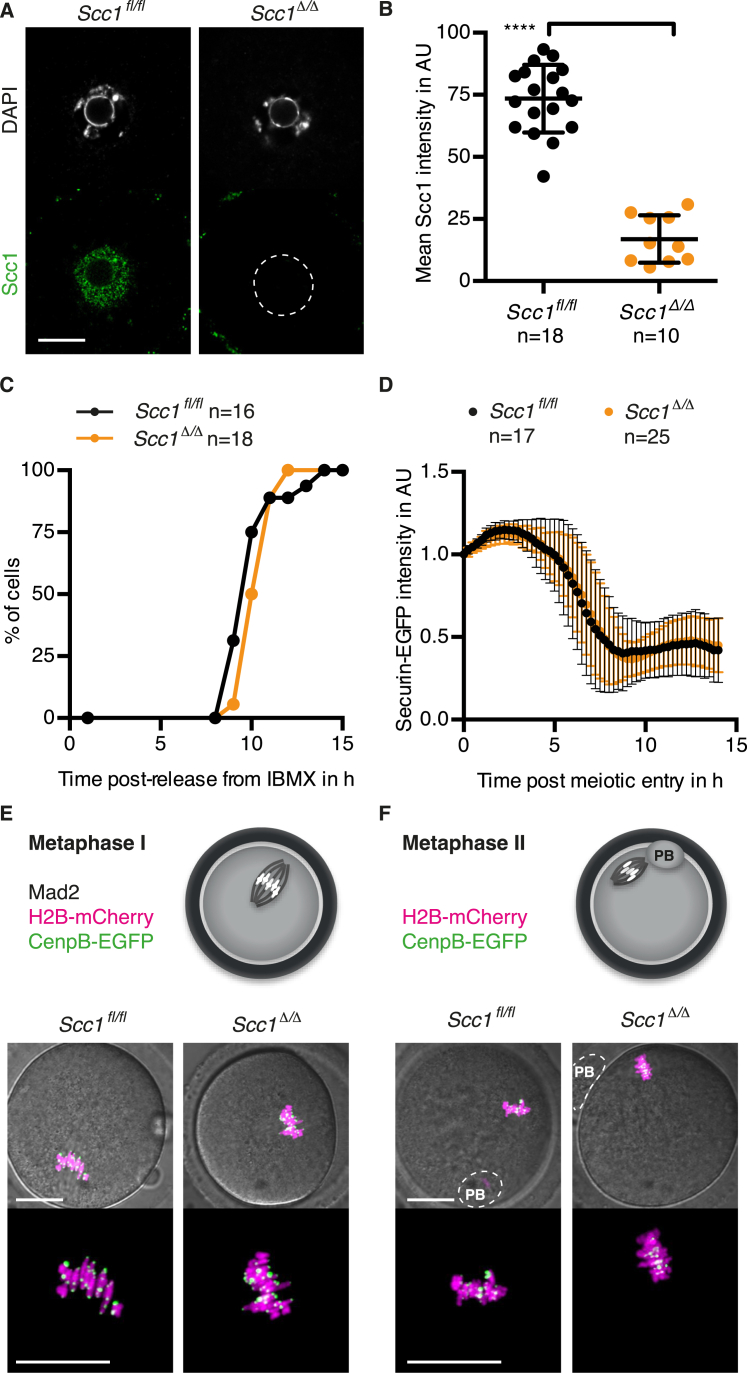
Scc1 Is Not Required for Oocytes to Grow to Maturity, Related to Figure 2 and Table S1 (A and B) Immunofluorescence detection of Scc1 in mature oocytes. (A) Representative images. (B) Quantification of mean nuclear Scc1 intensity. (C and D) Meiosis I kinetics of oocytes isolated from *Scc1*^*fl/fl*^ and *Scc1*^*fl/fl*^*(Tg)Zp3-Cre* females are assessed after release from phosphodiesterase inhibitor IBMX. (C) Kinetics of the first meiotic division (cytokinesis to extrude the first polar body). (D) Securin-EGFP intensity analysis during progression through meiosis I. Meiotic entry is scored according to nuclear envelope breakdown. (E and F) Representative live-cell still images of oocytes microinjected with mRNA of H2B-mCherry and CenpB-EGFP to visualize DNA and centromeres, respectively. (E) Arrest at metaphase I is achieved by Mad2 overexpression. (F) Naturally metaphase II arrested oocytes. n > 12 from > 2 females in each condition. ^∗∗∗∗^p < 0.0001, calculated using unpaired t test. All error bars indicate SD n, number of cells. AU, arbitrary units. PB, polar body. Scale bars, 20 μm.

To generate *Scc1* knockout oocytes, we utilized the same conditional knockout strategy as described above for *Xrcc1* (see Figure 1C). Genetic knockout of *Scc1* had no obvious effects on oocyte growth and mature *Scc1*^*fl/fl*^ and *Scc1*^*Δ/Δ*^ oocytes were isolated in comparable numbers (Table S1). Scc1 protein was efficiently depleted in *Scc1*^*Δ/Δ*^ oocytes (Figures S2A and S2B). To exclude that any defects observed after fertilization are due to defects accumulating in oocytes, we performed live-cell imaging of the first meiotic division of oocytes. The kinetics of APC/C activation and efficiency of polar body extrusion are similar in *Scc1*^*fl/fl*^ and *Scc1*^*Δ/Δ*^ oocytes (Figures S2C and S2D), suggesting that meiotic cell-cycle progression is unperturbed. No gross defects in chromosome alignment in metaphase I and metaphase II are detected in *Scc1*^*Δ/Δ*^ oocytes (Figures S2E and S2F), consistent with Rec8-cohesin maintaining sister chromatid cohesion (Tachibana-Konwalski et al., 2010). We conclude that Scc1 is not required for oocyte growth to maturity and the first meiotic division.

**Figure 2:**
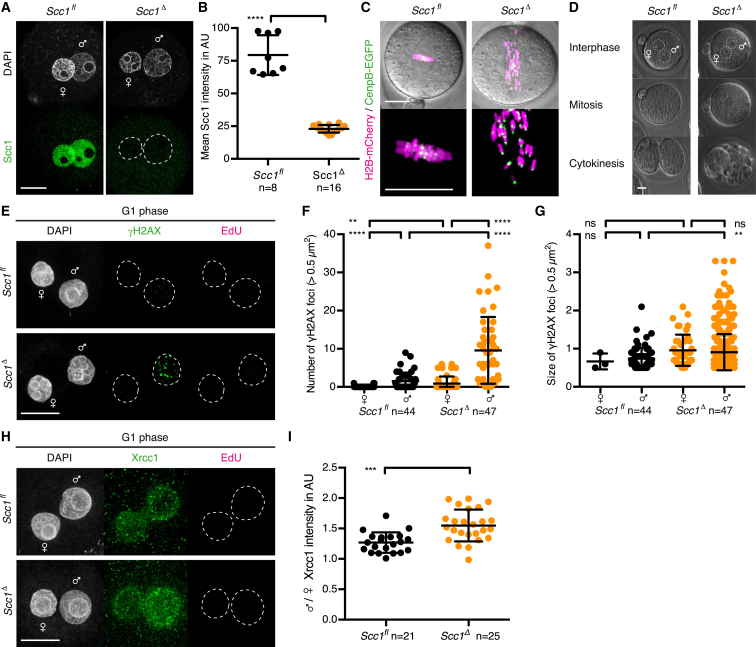
Absence of Scc1 Reveals Endogenous Paternal DNA Lesions in G1 Phase Zygotes (A and B) Immunofluorescence detection of Scc1 in zygotes. (A) Representative images. (B) Quantification of mean pronuclei Scc1 intensity. (C) Representative live-cell still images of metaphase arrested zygotes microinjected with mRNA of H2B-mCherry and CenpB-EGFP to visualize DNA and centromeres, respectively, and Mad2 to mediate metaphase arrest. n > 10 from less than two females in each condition. (D) Representative live-cell still images of *Scc1*^*fl*^ and *Scc1*^*Δ*^ zygotes at indicated stages. (E–G) Analysis of γH2AX foci in zygotes arrested in G1 phase by overexpression of hGeminin^L26A^ in mature oocytes followed by in vitro maturation and fertilization. Cells were cultured in continuous presence of EdU to verify inhibition of DNA replication and fixed at 9–10 hr post-fertilization. (E) Representative images. (F and G) Quantification of γH2AX foci (F) number and (G) size in maternal and paternal pronuclei, respectively. (H and I) Immunofluorescence detection of chromatin bound Xrcc1 in G1 phase zygotes. Cells were cultured in continuous presence of EdU from isolation until fixation to exclude cells that eventually started DNA replication. (H) Representative images. (I) Quantification of paternal to maternal ratio of mean Xrcc1 intensity. ^∗∗∗∗^p < 0.0001, ^∗∗^p < 0.01, ^∗∗∗^p < 0.001, calculated using unpaired t test (B and I) or Mann-Whitney test (F and G). All error bars indicate SD. n, number of cells. Scale bars, 20 μm. See also Tables S1 and S2 and Figures S2 and S3.

Scc1-cohesin is essential for sister chromatid cohesion in the first embryonic mitosis. To demonstrate that Scc1 depletion in zygotes (Figures 2A and 2B) causes precocious loss of cohesion, we examined mitotic chromosome alignment and kinetochores labeled with H2B-mCherry and CenpB-EGFP, respectively. As expected, a metaphase plate with bi-oriented sister kinetochores is assembled in *Scc1*^*fl*^ zygotes whereas single kinetochores associated with single chromatids track along the length of the spindle in *Scc1*^*Δ*^ zygotes (Figure 2C). These embryos occasionally divide to a highly aneuploid two-cell-like stage and fragment (Figure 2D). Consistent with these observations, *Scc1*^*fl/fl*^ *(Tg)Zp3-Cre* females are infertile. Therefore Scc1-cohesin inherited from the oocyte is essential for cohesion and viability after fertilization.

**Figure S3:**
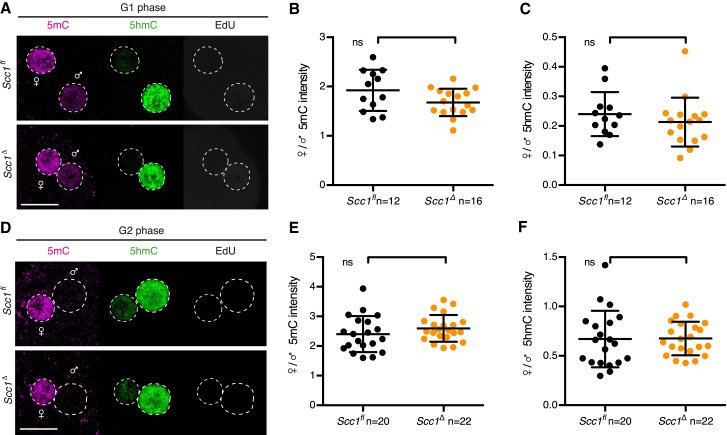
Analysis of 5mC and 5hmC in *Scc1*^fl^ and *Scc1*^Δ^ Zygotes, Related to Figure 2 and Table S2 (A–C) Immunofluorescence analysis of global 5mC and 5hmC in G1 phase zygotes. Cells were cultured in continuous presence of EdU from isolation until fixation to exclude cells that eventually started DNA replication. (A) Representative images. (B,C) Quantification of maternal to paternal ratio of mean (B) 5mC and (C) 5hmC intensity. (D–F) Immunofluorescence analysis of global 5mC and 5hmC in G2 phase zygotes. Cells were fixed after 30 min EdU pulse to exclude cells that still undergo DNA replication. (D) Representative images. (E,F) Quantification of maternal to paternal ratio of mean (E) 5mC and (F) 5hmC intensity. ^ns^ p > 0.5, calculated using unpaired t test. All error bars indicate SD n, number of cells. Scale bars, 20 μm.

Because cohesin is involved in DNA damage repair, we asked whether it is required to resolve paternal DNA lesions generated in G1 phase zygotes. Transient DNA lesions in G1 phase might be obscured by DNA breaks generated during DNA replication (Wossidlo et al., 2010). We therefore inhibited DNA replication by expressing a non-degradable geminin (hGeminin^L26A^), which prevents loading of the replicative MCM helicase (McGarry and Kirschner, 1998, Wohlschlegel et al., 2000, Wohlschlegel et al., 2002). In *Scc1*^*fl*^ zygotes, γH2AX is detectable at low levels in the paternal genome, consistent with efficient repair of DNA lesions (Figures 2E–2G). Remarkably, however, γH2AX foci persist in *Scc1*^*Δ*^ zygotes and are strongly enriched in the paternal genome (Figures 2E–2G), suggesting that cohesin is required for the efficient repair of paternal DNA lesions in G1 phase. This requirement must be independent of cohesin’s role in sister chromatid cohesion and HR because neither sister chromatids nor homologous chromosomes exist in these nuclei. We suggest cohesin’s role in organizing higher-order structure of chromatids may be required for the repair of paternal lesions (Fudenberg et al., 2016, Goloborodko et al., 2016). Paternal γH2AX foci are enlarged in *Scc1*^*Δ*^ zygotes (Figure 2G), suggesting either that cohesin restricts γH2AX spreading (Caron et al., 2012) or cohesin-mediated chromatin structure prevents foci clustering. The finding that cohesin is required for repairing paternal DNA lesions in G1 phase suggests that it affects BER. Consistent with this, Xrcc1 is enriched on paternal chromatin of *Scc1*^*Δ*^ compared to *Scc1*^*fl*^ zygotes (Figures 2H and 2I), implying that repair kinetics might be delayed in the absence of cohesin. Loss of 5mC and conversion to 5hmC are not globally affected in *Scc1*^*Δ*^ zygotes (Figure S3) as expected, because base excision is a late-step in DNA demethylation. Therefore, pre-replicative paternal DNA lesions require cohesin for repair in zygotes.

### Cohesin Is Required for Timely Entry into Mitosis in Zygotes

**Figure 3:**
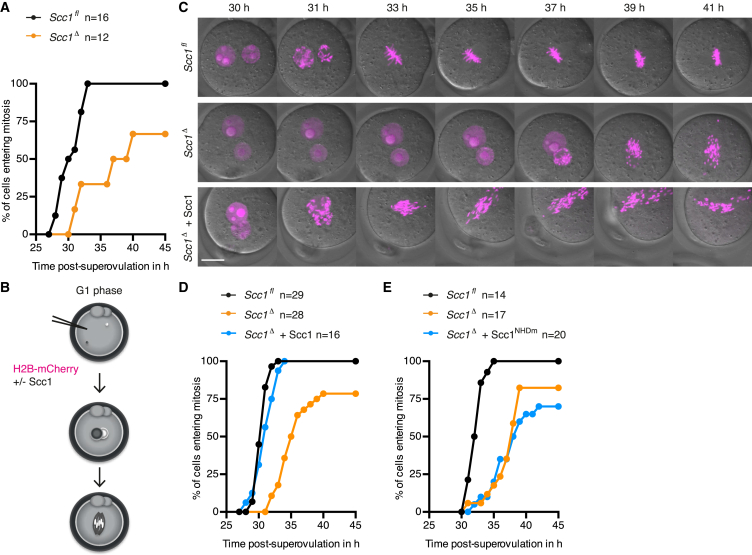
Scc1-Cohesin Promotes Timely Mitotic Entry of Zygotes Independent of Intact Sister Chromatid Cohesion but Dependent on Its Ability to Close the Scc1-Smc3 Interface (A) Mitotic entry kinetics of zygotes scored according to nuclear envelope breakdown. (B) Schematic of experimental approach to rescue kinetic defect of *Scc1*^*Δ*^ zygotes by microinjection of Scc1 mRNA into G1 phase. H2B-mCherry mRNA is used to visualize DNA. (C) Representative live-cell still images of zygotes microinjected with mRNA for H2B-mCherry ± Scc1. Mad2 is overexpressed to prolong mitosis for visualization of chromatids. Time in hr post-superovulation. (D and E) Kinetic rescue by reintroduction of (D) Scc1 mRNA or (E) Scc1 exit gate mutant (Scc1^NHDm^) mRNA. n, number of cells. Scale bars, 20 μm. See also Figure S4.

**Figure S4:**
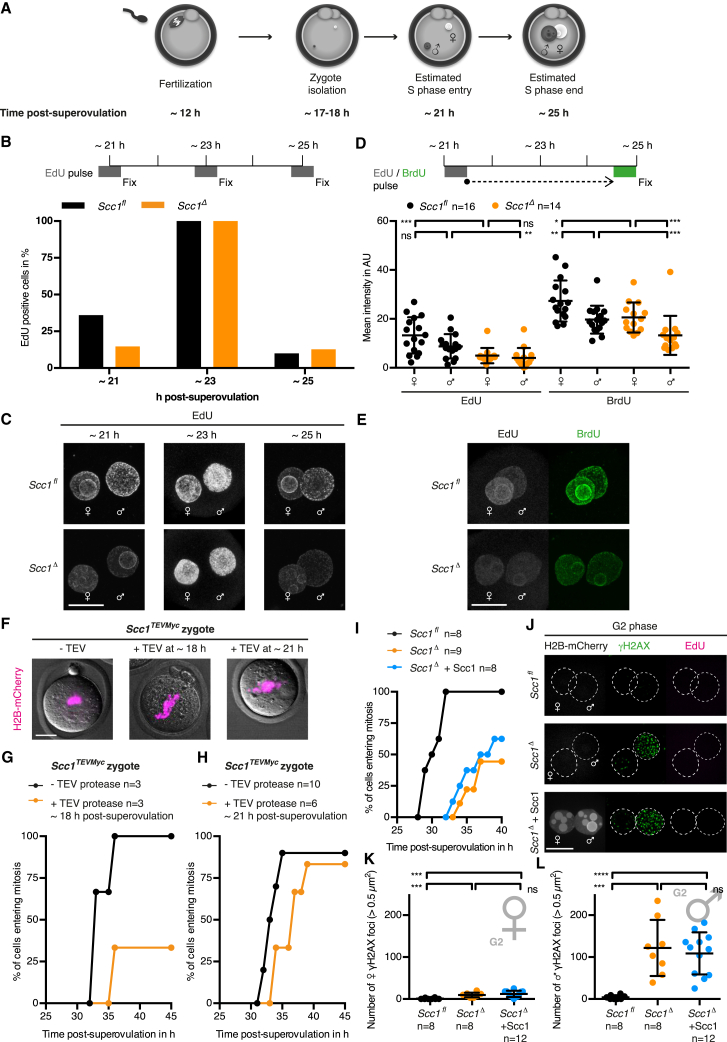
Analysis of *Scc1*^Δ^ Zygotes, Related to Figure 3 (A–E) S phase analysis of *Scc1*^*fl*^ and *Scc1*^*Δ*^ zygotes. (A) Schematic of experimental time course in relation to estimated S phase occurrence. (B and C) Analysis of S phase entry and end by using 30 min EdU pulses before fixation at indicated time post-superovulation. (B) Quantification of EdU positive cells at the indicated time points. (C) Representative images of EdU positive cells. Number of total cells analyzed in order of time points: *Scc1*^*fl*^ n = 25, 20, 60; *Scc1*^*Δ*^ n = 34, 24, 55. (D and E) Analysis of S phase duration by using an EdU/BrdU pulse chase approach. (D) A 30 min pulse of EdU was followed by a 3 hr wash out (dashed arrow) and a final 30 min BrdU pulse before fixation. Mean intensity of EdU and BrdU are given for maternal and paternal pronuclei, respectively. (E) Representative images. (F–H) Timely cell-cycle phase specific inactivation of Scc1 in zygotes utilizing TEV protease cleavage. (F) Representative images of metaphase *Scc1*^*TEVMyc*^ zygotes microinjected with H2B-mCherry. TEV protease mRNA was microinjected at defined time points post-superovulation which results in premature loss of sister chromatid cohesion. (G) Mitotic entry kinetics of *Scc1*^*TEVMyc*^ zygotes microinjected with TEV protease mRNA in early G1 phase (∼18 hr post-superovulation). (H) Mitotic entry kinetics of *Scc1*^*TEVMyc*^ zygotes microinjected with TEV protease mRNA at the G1/S phase boundary (∼21 hr post-superovulation). (I–L) Reintroduction of Scc1 mRNA into G2 phase zygotes. *Scc1*^*Δ*^ zygotes were microinjected with H2B-mCherry and wild-type Scc1 mRNA at 26 hr post-superovulation (G2 phase). Zygotes were directly incubated in EdU after microinjection to exclude cells that still undergo DNA replication. (I) Mitotic entry kinetics of zygotes scored according to nuclear envelope breakdown. (J-L) Immunofluorescence analysis of γH2AX foci in zygotes that are fixed after 2 hr EdU incubation that directly followed mRNA microinjection at 26 hr post-superovulation (G2 phase). Co-injection of H2B-mCherry mRNA is used as injection control. (J) Representative images. (K,L) Quantification of γH2AX foci number in (K) maternal and (L) paternal pronuclei. ^∗∗∗∗^p < 0.0001, ^∗∗∗^p < 0.001, ^∗∗^p < 0.01, ^∗^p < 0.1, ^ns^ p > 0.5, calculated using unpaired t test (D) or Mann-Whitney test (K,L). All error bars indicate SD n, number of cells. AU, arbitrary units. Scale bars, 20 μm.

We next asked whether paternal DNA lesions stabilized by cohesin loss affect cell-cycle progression. Using time-lapse microscopy, we observed an extended interphase arrest or delay in mitotic entry in *Scc1*^*Δ*^ but not in *Scc1*^*fl*^ zygotes (Figure 3A), suggesting that cohesin is required for timely entry into mitosis. This requirement appears to be specific for zygotes because cohesin is not known otherwise to be necessary for cell-cycle progression in interphase (Sonoda et al., 2001, Vagnarelli et al., 2004). Cohesin has been suggested to influence origin firing (Guillou et al., 2010). Because this is challenging to address in zygotes, we tested whether Scc1 affects S phase duration that might explain the extended interphase arrest. A pulse-chase experiment using BrdU and EdU revealed that DNA synthesis lasts for at least 4 hr and ceases with similar kinetics in *Scc1*^*fl*^ and *Scc1*^*Δ*^ zygotes (Figures S4A–S4E). Therefore, DNA replication timing is not grossly altered, although we cannot exclude that origin firing is affected. We note that the assays applicable to zygotes are not sensitive enough to demonstrate unequivocally that DNA replication is completed. Given that no active DNA synthesis is detected in arrested *Scc1*^*Δ*^ zygotes, we consider it most likely that the interphase arrest occurs in G2 phase.

It is conceivable that the interphase arrest of zygotes lacking Scc1 is due to changes in gene expression in oocytes rather than a direct requirement for cohesin in zygotes. To exclude pleiotropic effects, we performed two experiments. First, we utilized a mouse strain *Scc1*^*TEVMyc/TEVMyc*^ in which all Scc1 is cleavable by tobacco etch virus (TEV) protease (Tachibana-Konwalski et al., 2010). The advantage of this approach is that oocyte development proceeds in the presence of Scc1 and microinjection of TEV protease into zygotes destroys cohesin in a cell-cycle phase-specific manner. *Scc1*^*TEVMyc*^ zygotes expressing TEV protease in early G1 phase but not in G1/S phase recapitulate delayed entry into mitosis (Figures S4F–S4H), suggesting that the delay is due to a requirement for Scc1 in zygotes and not a pleiotropic consequence of lacking Scc1 in oocytes. The complementary approach tested whether expressing Scc1 in *Scc1*^*Δ*^ zygotes rescues the kinetics of mitotic entry. G1 phase zygotes were microinjected with mRNA encoding H2B-mCherry and with or without mRNA encoding Scc1 (Figure 3B). Mad2 was coexpressed to activate the spindle assembly checkpoint and prolong prometaphase in order to better visualize sister versus single chromatids (Wassmann et al., 2003). Remarkably, *Scc1*^*Δ*^ zygotes expressing Scc1 enter mitosis with kinetics that are indistinguishable from *Scc1*^*fl*^ zygotes (Figures 3C and 3D). The kinetic rescue of mitotic entry demonstrates that the interphase arrest is a direct consequence of lacking Scc1 in zygotes. Interestingly, the timing of mitotic entry does not depend on intact sister chromatid cohesion because *Scc1*^*Δ*^ zygotes expressing Scc1 display single chromatids in mitosis (Figure 3C), which could be due to insufficient expression of Scc1 during DNA replication. It is nevertheless conceivable that some cohesion established in S phase contributes to the rescue of mitotic entry because expressing Scc1 in G2 phase *Scc1*^*Δ*^ zygotes has little or no effect (Figures S4I–S4L). To test whether the kinetic rescue depends on the cohesin ring, we expressed an Scc1 exit gate mutant (Scc1^NHDm^) (Gligoris et al., 2014, Huis in ’t Veld et al., 2014). Scc1^NHDm^ eliminates cohesin’s ability to close the Scc1-Smc3 interface, resulting in an open cohesin form that cannot stably associate to chromatin (Huis in ’t Veld et al., 2014). Unlike wild-type Scc1, expression of Scc1^NHDm^ in *Scc1*^*Δ*^ G1 phase zygotes does not restore timely mitotic entry (Figure 3E). We conclude that cohesin’s ability to form a closed ring structure is required for timely entry into mitosis in zygotes.

### Scc1 Is Required for Timely Mitotic Entry in a Paternal Genome- and Tet3-Dependent Manner

**Figure 4:**
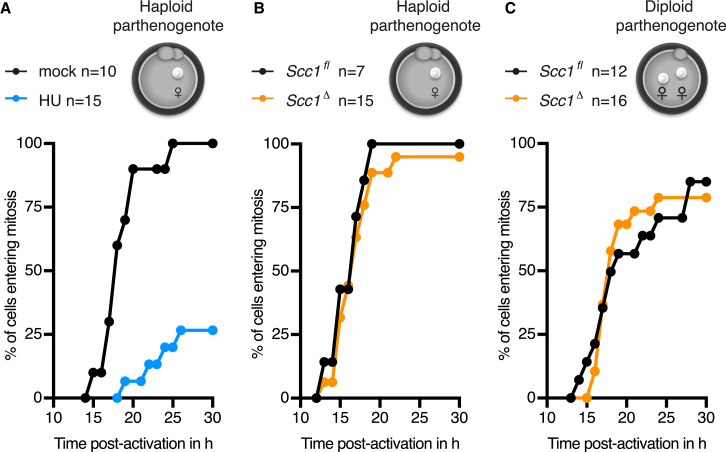
Scc1 Is Involved in Paternal Genome-Related Processes that Drive Timely Progression of the First Embryonic Cell Cycle (A–C) Mitotic entry kinetics of (A) haploid wild-type parthenogenotes treated with hydroxyurea (HU) (B) haploid *Scc1*^*fl*^ and *Scc1*^*Δ*^ parthenogenotes and (C) diploid *Scc1*^*fl*^ and *Scc1*^*Δ*^ parthenogenotes. n, number of cells.

If loss of Scc1 delays timely mitotic entry because unrepaired paternal DNA lesions signal to the cell-cycle machinery, then we would predict that preventing paternal chromatin reprogramming would render Scc1 dispensable for cell-cycle progression. We therefore tested whether parthenogenetic embryos containing a maternal but no paternal genome enter mitosis in a timely fashion, irrespective of Scc1. Because little is known about checkpoint activation in parthenogenetic embryos, we tested whether hydroxyurea treatment, which leads to depletion of the dNTP pool, activates an S phase checkpoint, and causes an interphase arrest. Indeed, 75% of cells arrested in interphase and the remaining cells entered mitosis with severely delayed kinetics (Figure 4A). Therefore, parthenogenetic embryos are capable of mounting a checkpoint response.

To test whether Scc1 is required for timely mitotic entry in a paternal genome-dependent manner, we examined cell-cycle progression of *Scc1*^*fl*^ and *Scc1*^*Δ*^ haploid parthenogenotes. Remarkably, both parthenogenotes with or without Scc1 enter mitosis with the same kinetics (Figure 4B). This suggests that Scc1 is not required for timely mitotic entry in the absence of a paternal genome, even though parthenogenotes are capable of mounting a checkpoint response. To exclude that checkpoint activation in the absence of Scc1 depends on ploidy, we examined *Scc1*^*fl*^ and *Scc1*^*Δ*^ diploid parthenogenotes harboring two maternal genomes and found little to no difference in the kinetics of mitotic entry (Figure 4C). This demonstrates that Scc1 is not required for cell-cycle progression per se and implies that it is particularly important for paternal genome-related molecular processes.

**Figure 5:**
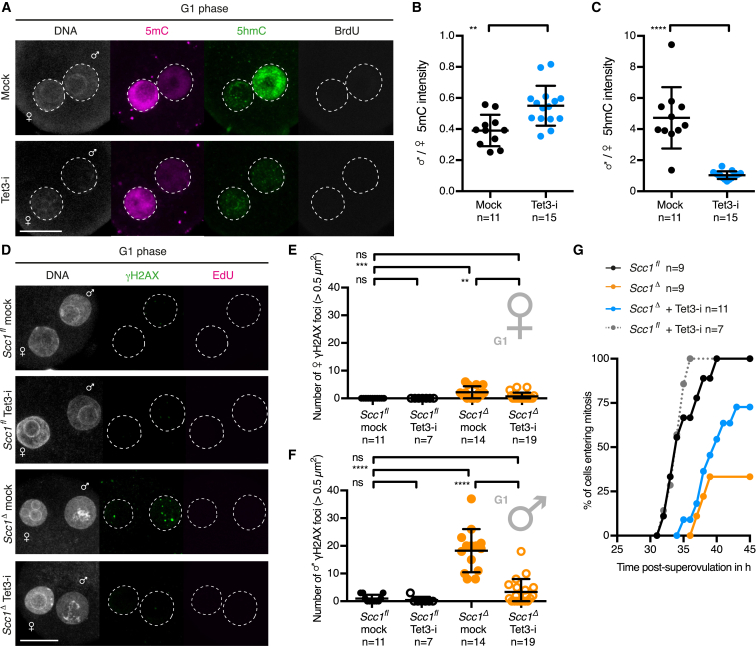
Tet3 Contributes to Zygotic Checkpoint Activation Uncovered by Lack of Scc1 (A–C) Immunofluorescence analysis of global 5mC and 5hmC in G1 phase wild-type zygotes treated with Tet3 inhibitor (Tet3-i). (A) Representative images. (B and C) Quantification of paternal to maternal ratio of mean (B) 5mC and (C) 5hmC intensity. (D–F) Analysis of G1 phase γH2AX foci in zygotes treated with Tet3 inhibitor (Tet3-i). (D) Representative images. (E and F) Quantification of γH2AX foci number in (E) maternal and (F) paternal pronuclei. (G) Analysis of mitotic entry kinetics according to nuclear envelope breakdown of zygotes treated from G1 phase onward with Tet3 inhibitor (Tet3-i). Note for (A)–(F), cells were fixed after 1 hr incubation in presence of BrdU or EdU to exclude cells that eventually started DNA replication. ^∗∗∗∗^p < 0.0001, ^∗∗∗^p < 0.001, ^∗∗^p < 0.01, ^ns^p > 0.5, calculated using unpaired t test (B and C) or Mann-Whitney test (E and F). All error bars indicate SD. n, number of cells. Scale bars, 20 μm.

To test whether the critical paternal genome-related process is Tet3-driven DNA demethylation, we modulated Tet3 activity in zygotes. Pharmacological inhibition of Tet3 slightly affected 5mC levels and considerably prevented the accumulation of 5hmC in paternal chromatin of G1 phase zygotes (Figures 5A–5C). Further, we found that Tet3 inhibition leads to a significant reduction of paternal γH2AX foci in G1 phase *Scc1*^*Δ*^ zygotes (Figures 5D–5F). This suggests that Tet3-dependent activity, presumably via DNA demethylation, generates paternal DNA lesions that require Scc1 for repair. Tet3 inhibition improved the efficiency of mitotic entry of *Scc1*^*Δ*^ zygotes (Figure 5G) but did not fully abrogate checkpoint activation, perhaps due to residual Tet3 activity or accumulation of Tet3-independent DNA lesions in *Scc1*^*Δ*^ zygotes. Together, these findings suggest that Tet3-dependent paternal DNA lesions signal to the cell-cycle machinery and contribute to checkpoint activation.

### Timely Mitotic Entry Requires DNA Repair-Proficient Cohesin

**Figure S5:**
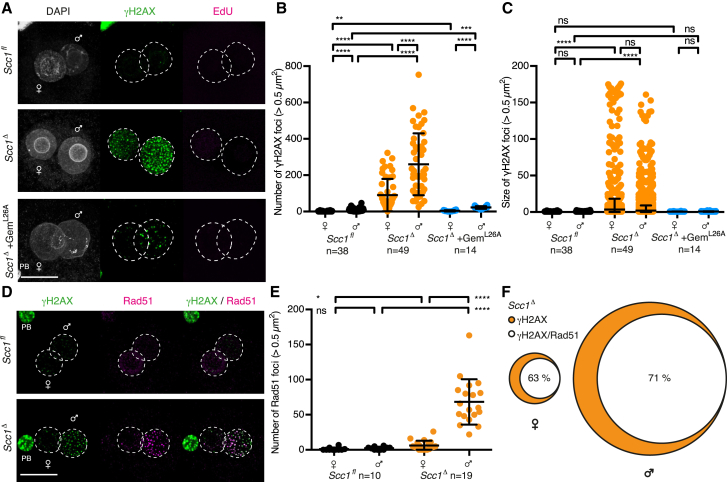
Zygotic γH2AX Foci Accumulate during Cell-Cycle Progression in Absence of Scc1, Related to Figure 6 (A–C) Immunofluorescence analysis of γH2AX foci in G2 phase zygotes. (A) Representative images. The upper two panels display zygotes fixed after 30 min EdU pulse at ∼14 hr post-fertilization to exclude cells that still undergo DNA replication. The lowest panel shows a zygote obtained by overexpression of hGeminin^L26A^ in mature oocytes followed by in vitro maturation and fertilization; these cells were cultured in continuous presence of EdU and fixed at ∼14 hr post-fertilization. (B,C) Quantification of γH2AX foci (B) number and (C) size in maternal and paternal pronuclei, respectively. (D–F) Immunofluorescence analysis of co-occurring Rad51 and γH2AX foci in G2 phase zygotes. Zygotes are pre-extracted and fixed after 30 min EdU pulse (not shown). (D) Representative images. (E) Quantification of Rad51 foci number in maternal and paternal pronuclei, respectively. (F) Venn diagram of γH2AX foci overlapping with Rad51 signals in maternal and paternal pronuclei of *Scc1*^*Δ*^ zygotes (n = 19). ^∗∗∗∗^p < 0.0001, ^∗∗∗^p < 0.001, ^∗^p < 0.1, ^ns^ p > 0.5, calculated using Mann-Whitney test. All error bars indicate SD n, number of cells. PB, polar body. Scale bars, 20 μm.

To better understand why Tet3 inhibition did not fully rescue mitotic entry, we investigated whether there might be other DNA lesions accumulating during progression through the cell cycle in the absence of cohesin. We therefore analyzed γH2AX foci after G1 phase. Few, if any, γH2AX foci are detected by immunofluorescence in paternal and maternal pronuclei of G2 phase *Scc1*^*fl*^ zygotes (Figures S5A–S5C). In contrast, many γH2AX foci accumulate on both maternal and paternal chromatin in G2 phase *Scc1*^*Δ*^ zygotes, with significantly more foci present in the paternal genome (Figures S5A–S5C). The maternal genome does not undergo extensive DNA demethylation at this stage (see Figure S3). However, the increase in γH2AX foci in *Scc1*^*Δ*^ maternal pronuclei after S phase suggests that lesions accumulate from DNA replication stress in the absence of cohesin on both parental genomes (Guillou et al., 2010), providing a possible explanation for why Tet3 inhibition did not fully alleviate checkpoint activity. If the accumulation of DNA lesions in absence of cohesin depends on DNA replication stress, then we would expect that inhibiting DNA replication should prevent accumulation of additional lesions. Consistent with this, arrested *Scc1*^*Δ*^ zygotes expressing hGeminin^L26A^ display nearly no maternal and few paternal γH2AX foci (Figures S5A–S5C). DNA lesions that arise due to replication stress might be expected to recruit Rad51 for HR-mediated repair (Lundin et al., 2003, Sonoda et al., 1998). We observe some γH2AX foci overlapping with Rad51 and other γH2AX foci without detectable Rad51 in *Scc1*^*Δ*^ zygotes (Figures S5D–S5F). We cannot distinguish whether the two populations reflect distinct DNA damage sites or whether the detection threshold of Rad51 is limiting. Overall, we conclude that cohesin is required to resolve DNA lesions at both maternal and paternal genomes of zygotes.

**Figure S6:**
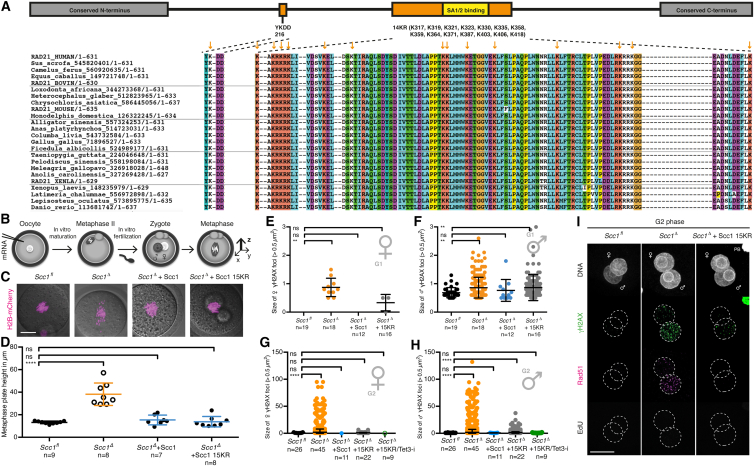
Analysis of Scc1 15KR, Related to Figure 6 (A) Conservation of the 15 lysines mutated in Scc1 15KR. Schematic of mouse Scc1 domains is shown on top. Orange indicates regions containing lysines mutated in Scc1 15KR according to Wu et al. (2012). (B–D) Rescue of precocious sister chromatid cohesion loss in *Scc1*^*Δ*^ zygotes using Scc1 15KR. (B) Schematic of experimental approach to rescue sister chromatid cohesion by microinjection of Scc1 and H2B-mCherry mRNA into unfertilized oocytes with subsequent in vitro maturation and fertilization. (C) Representative live-cell still images of zygotes at metaphase, which corresponds to one time point before anaphase. Scale bars, 20 μm. (D) Quantification of metaphase plate height (z in (B)). Filled dots indicate cells with sister chromatids, while open dots represent cells with single chromatids. (E–H) Size of γH2AX foci in *Scc1*^*Δ*^ zygotes expressing Scc1 15KR. Rescue of γH2AX foci in *Scc1*^*Δ*^ zygotes was performed by microinjection of mRNA for Scc1 (wild-type or Scc1 15KR) into oocytes with subsequent in vitro maturation and fertilization. Tet3 inhibitor (Tet3-i) was added at 4 hr post-fertilization. (E,F) Analysis of γH2AX foci size in (E) maternal and (F) paternal pronuclei of in G1 phase fixed zygotes. Relates to Figures 6A–6C. (G,H) Analysis of γH2AX foci size in (G) maternal and (H) paternal pronuclei of in G2 phase fixed zygotes. Relates to Figures 6D–6F. (E) Representative images of pre-extracted G2 phase zygotes with immunofluorescence detection of γH2AX and Rad51. Microinjection of Scc1 15KR mRNA was done into *Scc1*^*Δ/Δ*^ oocytes with subsequent in vitro maturation and fertilization. Cells are fixed after 30 min EdU pulse to exclude cells that still undergo DNA replication. n > 6. ^∗∗∗∗^p < 0.0001, ^∗∗^p < 0.01, ^ns^ p > 0.5, calculated using unpaired t test (D) or Mann-Whitney test (E-H). All error bars indicate SD n, number of cells.

To study exclusively the paternal DNA lesions arising from DNA demethylation in G1 phase, we established an in vivo structure function assay to rescue sister chromatid cohesion but not DNA repair-related functions of cohesin. The expression of a version of Scc1 that is cohesion-proficient but DNA repair-deficient should prevent the formation of maternal and paternal DNA lesions generated by replication stress, but not of paternal lesions generated by zygotic reprogramming. The Mms21 SUMO-ligase is required for efficient DNA repair and sumoylates Scc1 during the DNA damage response (Wu et al., 2012). Substitution of 15 lysine residues conserved among metazoan Scc1 (Figure S6A) renders Scc1 15KR defective for DNA damage repair in tissue culture cells but maintains its function in sister chromatid cohesion (Wu et al., 2012).

**Figure 6:**
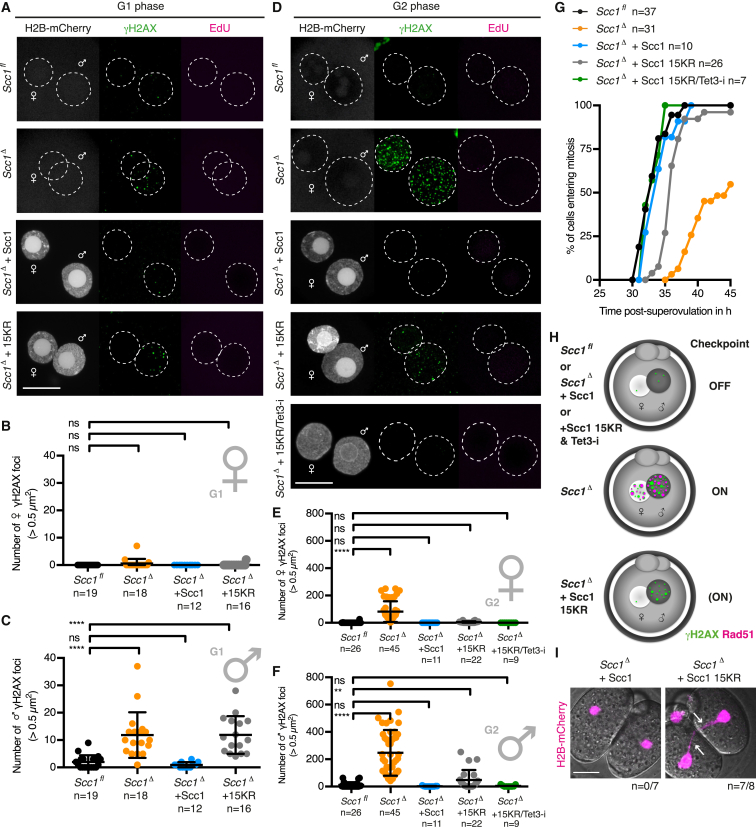
In Vivo Structure Function Assay Using Scc1 15KR Demonstrates the Existence of Tet3-Dependent Paternal DNA Lesions that Trigger Zygotic Checkpoint Response (A–C) G1 phase γH2AX foci in *Scc1*^*Δ*^ zygotes expressing wild-type Scc1 or Scc1 15KR mRNA. Cells were fixed after 1 hr incubation in presence of EdU to exclude cells that eventually started DNA replication. (A) Representative images. (B and C) Quantification of γH2AX foci number in (B) maternal and (C) paternal pronuclei. (D–F) Immunofluorescence analysis of γH2AX foci in G2 phase zygotes that are fixed after 30 min EdU pulse to exclude cells that still undergo DNA replication. The Tet3 inhibitor (Tet3-i) was added 4 hr post-fertilization. (D) Representative images. (E and F) Quantification of γH2AX foci number in (E) maternal and (F) paternal pronuclei. (G) Mitotic entry kinetics of zygotes scored according to nuclear envelope breakdown. Microinjection of wild-type Scc1 or Scc1 15KR mRNA was performed in G1 phase. (H) Illustration of γH2AX foci populations and their relation to checkpoint activation. (I) Representative live-cell still images at anaphase of *Scc1*^*Δ*^ zygotes expressing either wild-type Scc1 or Scc1 15KR. Arrows indicate chromatin bridges. Occurrence of anaphase bridges per total cell number is given. Note for (A)–(F) and (I) wild-type Scc1 or Scc1 15KR mRNA was microinjected in oocytes with subsequent in vitro maturation and fertilization. Co-injection of H2B-mCherry mRNA is used as injection control. ^∗∗∗∗^p < 0.0001, ^∗∗^p < 0.01, ^ns^p > 0.5, calculated using Mann-Whitney test. All error bars indicate SD. n, number of cells. Scale bars, 20 μm. See also Figures S5 and S6.

First, we tested if wild-type Scc1 expressed in *Scc1*^*Δ/Δ*^ oocytes followed by in vitro fertilization rescues cohesion in *Scc1*^*Δ*^ zygotes (Figure S6B). Sister chromatids align on a metaphase plate with ∼13 μm in height for *Scc1*^*fl*^ zygotes (Figures S6C and S6D). Loss of cohesion in *Scc1*^*Δ*^ zygotes results in a broad metaphase plate that spans up to 60 μm (Figures S6C and S6D). Expressing either wild-type Scc1 or Scc1 15KR in *Scc1*^*Δ*^ zygotes restores the metaphase plate to a similar height as in *Scc1*^*fl*^ zygotes (Figures S6C and S6D), indicating that Scc1 15KR established cohesion. On the other hand, if this mutant is defective for DNA damage repair, then we would expect that it cannot rescue paternal DNA lesions in G1 phase of *Scc1*^*Δ*^ zygotes. To test this, *Scc1*^*Δ/Δ*^ oocytes were microinjected with mRNAs encoding H2B-mCherry and wild-type or Scc1 15KR. We found that Scc1, but not Scc1 15KR, rescues γH2AX foci of paternal pronuclei of *Scc1*^*Δ*^ zygotes (Figures 6A–6C, S6E, and S6F). Therefore, Scc1 15KR is proficient for cohesion but defective in repairing paternal DNA lesions in G1 phase.

Using the in vivo structure-function rescue assay, we aimed to test whether the paternal DNA lesions generated by Tet3 activity activate the checkpoint in the absence of maternal DNA lesions. We therefore performed a series of rescue experiments by expressing Scc1 and assaying γH2AX foci in G2 phase. Expressing wild-type Scc1 in *Scc1*^*Δ*^ zygotes results in no detectable γH2AX foci (Figures 6D–6F, S6G, and S6H), demonstrating that both maternal and paternal DNA lesions are prevented or repaired. Expressing Scc1 15KR resulted in accumulation of γH2AX foci in paternal but not in maternal DNA, consistent with this mutant being defective in DNA repair. These paternal DNA lesions do not recruit Rad51 (Figure S6I), implying that they were not generated due to replication stress. Importantly, expressing Scc1 15KR and inhibiting Tet3 prevents all paternal DNA lesions in *Scc1*^*Δ*^ G2 phase zygotes (Figures 6D–6F). This provides strong evidence that persisting paternal DNA lesions are due to Tet3-dependent reprogramming in G1 phase (see Figure 5).

We now turned to the key question of whether checkpoint activation depends on Tet3-dependent paternal DNA lesions. If unrepaired paternal DNA lesions are sufficient to activate a checkpoint, then we would expect that Scc1 15KR delays entry into mitosis. Indeed, *Scc1*^*Δ*^ zygotes expressing Scc1 15KR continue to display delayed mitotic entry, although less severe than uninjected *Scc1*^*Δ*^ zygotes that also accumulate maternal DNA lesions. If the unrepaired paternal DNA lesions due to Tet3-dependent reprogramming trigger checkpoint activation, then Tet3 inhibition in Scc1 15KR-expressing zygotes should prevent checkpoint activation. Crucially, Tet3 inhibition in Scc1 15KR zygotes leads to timely mitotic entry comparable to *Scc1*^*fl*^ controls (Figures 6G and 6H), suggesting that no checkpoint has been activated. We therefore conclude that Tet3-dependent zygotic reprogramming generates paternal DNA lesions that are monitored by a surveillance mechanism and activate a checkpoint that prevents entry into mitosis.

We addressed the consequence for the embryo if these DNA lesions are not repaired in a timely fashion. Cells entering mitosis with DNA damage frequently display anaphase bridges that can lead to chromosome fragmentation and aneuploidy (Burrell et al., 2013, Chan et al., 2009). While no anaphase bridges are detected in *Scc1*^*Δ*^ zygotes expressing Scc1, 7/8 *Scc1*^*Δ*^ zygotes expressing Scc1 15KR display anaphase bridges and subsequently fragment (Figures 6I). Therefore, timely repair of paternal DNA lesions is important for faithful chromosome segregation and embryonic development.

### A Chk1-Dependent Checkpoint Sensitive to IVF Culturing Conditions Prevents Entry into Mitosis

**Figure 7:**
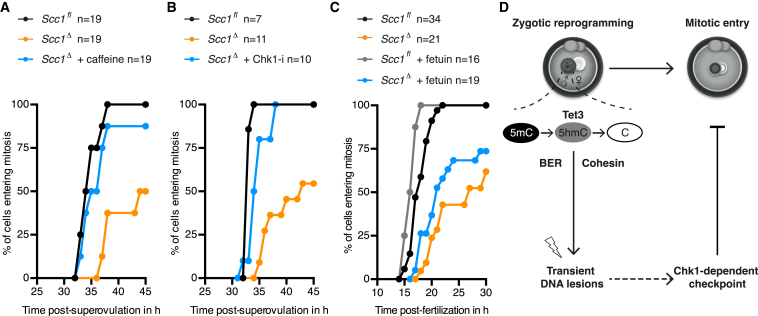
Chk1 Mediates the Zygotic Checkpoint Uncovered by Lack of Scc1 (A–C) Mitotic entry kinetics according to nuclear envelope breakdown. (A and B) Zygotes treated from G1 phase onward with specific inhibitors against (A) Atr/Atm/DNA-PKcs (caffeine) and (B) Chk1 (Chk1-i, PF-477736). (C) Zygotes obtained by in vitro fertilization analyzed in presence or absence of fetuin. (D) Zygotic reprogramming of the paternal genome includes Tet3-dependent conversion of 5mC to 5hmC and involves BER proteins and cohesin to repair accompanying transient DNA lesions. Stabilization of paternal DNA lesions generated during reprogramming can activate a checkpoint that potentially links completion of reprogramming with mitosis. n, number of cells. See also Figure S7.

**Figure S7:**
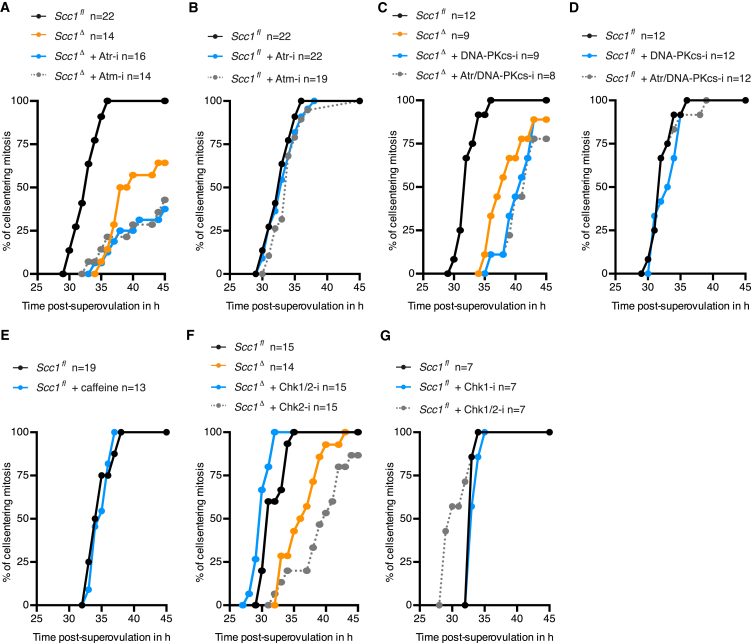
Treatment of Zygotes with Specific Kinase Inhibitors, Related to Figure 7 Analysis of mitotic entry kinetics scored according to nuclear envelope breakdown of zygotes treated from G1 phase onward with specific kinase inhibitors against: (A and B) Atr (Atr-i, VE-821) or Atm (Atm-i, KU-55933), (C and D) DNA-PKcs (DNA-PKcs-i, NU7026) or simultaneously Atr/DNA-PKcs (VE-821/NU7026), (E) Atr/Atm/DNA-PKcs (caffeine), See also Figure 7H, (F) simultaneously Chk1/Chk2 (Chk1/2-i, AZD7762) or Chk2 (Chk2-i, NSC109555), (G) simultaneously Chk1/Chk2 (Chk1/2-i, AZD7762) or Chk1 (Chk1-i, PF-477736). n, number of cells.

Given that paternal DNA lesions in *Scc1*^Δ^ zygotes are sufficient to cause an interphase arrest and therefore presumably activate a cell-cycle checkpoint, we tested whether molecular players of the DNA damage checkpoint are involved. We probed whether the two major DDR transducer kinases, namely ataxia-telangiectasia mutated (Atm) and ataxia telangiectasia and Rad3 related (Atr) (Harper and Elledge, 2007), facilitate the interphase arrest in *Scc1*^*Δ*^ zygotes. If so, then chemical inhibition of these kinases should bypass the arrest and rescue kinetics of mitotic entry. We found that inhibition of Atm or Atr does not relieve the cell-cycle delay in *Scc1*^*Δ*^ zygotes (Figures S7A and S7B). DNA-dependent protein kinase catalytic subunit (DNA-PKcs) can activate a checkpoint under conditions of Atr inhibition and replicative stress (Buisson et al., 2015). However, the interphase arrest in *Scc1*^Δ^ zygotes was not bypassed upon inhibition of DNA-PKcs alone or in combination with Atr (Figures S7C and S7D). To simultaneously inhibit Atm, Atr, and DNA-PKcs, *Scc1*^*Δ*^ zygotes were incubated with caffeine, which triggered mitotic entry of *Scc1*^*Δ*^ zygotes with similar kinetics as in *Scc1*^fl^ zygotes (Figures 7A and S7E). Therefore, the three DDR kinases Atm, Atr, and DNA-PKcs, likely act redundantly to activate a cell-cycle checkpoint in response to endogenous DNA lesions in zygotes.

DNA damage checkpoints are mediated by the structurally unrelated serine/threonine kinases Chk1 and Chk2 that act downstream of DDR transducer kinases (Harper and Elledge, 2007). To identify which effector kinase mediates the cell-cycle checkpoint in *Scc1*^*Δ*^ zygotes, we tested whether chemical inhibition of Chk1 and/or Chk2 rescues kinetics of mitotic entry. Inhibition of Chk1 alone, but not Chk2 alone, bypasses the interphase arrest and rescues kinetics of mitotic entry of *Scc1*^*Δ*^ zygotes (Figures 7B, S7F, and S7G). We conclude that unrepaired DNA lesions, particularly in the paternal genome, activate a Chk1-dependent checkpoint that coordinates DNA repair with mitosis.

The discovery of a Chk1-dependent checkpoint that coordinates zygotic reprogramming and mitotic entry has an important implication for in vitro fertilization. Current practice includes transplanting embryos that divide fastest in culture. However, our studies suggest that an early division might not necessarily reflect the best quality embryo because it could reflect weak checkpoint activation. Therefore, we investigated whether the Chk1-dependent checkpoint is activated efficiently in embryos produced by in vitro fertilization. We find that like naturally produced embryos, in vitro fertilized *Scc1*^*Δ*^ zygotes activate a checkpoint that delays entry into mitosis (Figure 7C). Bovine, and sometimes mouse, in vitro fertilizations are carried out in the presence of fetuin to increase the fertilization efficiency (Dietzel et al., 2013, Landim-Alvarenga et al., 2002). Unexpectedly, we found that fetuin leads to faster kinetics of mitotic entry (Figure 7C), suggesting that the checkpoint was attenuated under these conditions. We therefore propose that culturing conditions during in vitro fertilization may play a critical role in potentiating the checkpoint that monitors zygotic reprogramming.

## Discussion

How reprogramming to totipotency is achieved within one cell cycle is poorly understood. Our study has uncovered a Chk1-dependent checkpoint that responds to endogenous reprogramming-dependent DNA lesions in zygotes and coordinates DNA repair with mitosis. This is based on the unexpected finding that Scc1 is required for timely progression through the first cell cycle in a paternal genome-dependent manner. Currently there are only two genes known to be essential for progression from the one- to two-cell embryo, namely the maternal mRNA regulator Zar1 (Wu et al., 2003, Yamamoto et al., 2013) and the histone chaperone Hira (Lin et al., 2014), neither of which are related to DNA damage repair. The requirement for DNA repair-proficient cohesin in embryos with but not without a paternal genome supports the hypothesis that active DNA demethylation occurring at paternal chromatin involves a DNA repair-coupled mechanism (Hajkova et al., 2010, Wossidlo et al., 2010).

Paternal DNA lesions generated during G1 phase, when the paternal genome is undergoing active DNA demethylation, have been challenging to study because they are transiently detected and thereafter possibly obscured by DNA breaks caused by replication stress (Wossidlo et al., 2010). We have been able to study these paternal DNA lesions by utilizing conditional cohesin knockout zygotes in which DNA lesions persist into G2 phase. The paternal DNA lesions activate a cell-cycle checkpoint in a dose-dependent manner. We have identified several molecular players of the checkpoint and find that Tet3 activity is necessary for checkpoint activation.

The finding that zygotes activate a checkpoint in response to unrepaired DNA lesions in the absence of cohesin is distinct from observations in somatic cells. Indeed, cohesin has been implicated in intra-S and G2/M checkpoints in response to DNA damage (Kim et al., 2002, Kitagawa et al., 2004, Watrin and Peters, 2009, Yazdi et al., 2002). One difference lies in the signaling modes utilized in zygotes and mammalian cells lacking cohesin. Cohesin was shown to be required for complete activation of Chk2 in response to DNA damage in mammalian cells (Watrin and Peters, 2009). Conversely, the surveillance mechanism in zygotes involves Chk1 activation and is cohesin-independent. Additionally, the type of DNA damage is different in these contexts, as the previous studies utilized ionizing radiation (Kim et al., 2002, Kitagawa et al., 2004, Watrin and Peters, 2009, Yazdi et al., 2002), and our work in zygotes examines endogenous DNA lesions. It is therefore conceivable that different branches of DDR are activated depending on the type of DNA lesion.

Finally, it is tempting to speculate that the checkpoint monitors zygotic reprogramming and coordinates the repair of reprogrammed loci with mitosis. Deeper insights into the mechanism of active DNA demethylation are necessary to fully understand the surveillance mechanism. Nevertheless, we provide evidence of a DDR-mediated checkpoint signaling that responds to endogenous DNA lesions during reprogramming to totipotency in zygotes. We demonstrate that the paternal DNA lesions are due to Tet3 activity, and we can exclude that they arise from DNA damage brought in by sperm. Whether the effect of Tet3 activity on DNA lesions is solely due to DNA demethylation via modification of 5mC or possible roles in transcription remains to be determined. Our work implies that Scc1-cohesin accumulating in oocytes is crucial after fertilization not only for sister chromatid cohesion but also for repairing endogenous DNA lesions. Failure to do so can result in a one-cell arrest or fragmented embryo, which will manifest as embryo loss and infertility. These findings highlight the importance of DNA damage repair mechanisms at the oocyte-to-zygote transition and are relevant for assisted reproductive and induced pluripotent stem cell technologies.

## STAR★Methods

### Key Resources Table

**Table:** 

REAGENT or RESOURCE	SOURCE	IDENTIFIER
**Antibodies**		

Anti-5hmC	Active Motif	Cat#39769
Anti-5mC	Eurogentec	Cat#BI-MECY-0100; RRID: AB_2616058
Anti-BrdU	Abcam	Cat#ab6326; RRID: AB_305426
Anti-Rad51	Santa Cruz	Cat#sc-8349; RRID: AB_2253533
Anti-Rad21 (anti-Scc1)	Millipore	Cat#05-908; RRID: AB_417383
Anti-Xrcc1	Serotec	Cat#AHP832; RRID: AB_2218473
Anti-γH2AX	Abcam	Cat#ab22551; RRID: AB_447150
Alexa Fluor 488 Goat Anti-Mouse IgG (H+L)	Invitrogen	Cat#A-11029
Alexa Fluor 568 Goat Anti-Mouse IgG (H+L)	Invitrogen	Cat#A-11031
Oregon Green 488 Goat Anti-Rabbit IgG (H+L)	Invitrogen	Cat#O-6381
Alexa Fluor 568 Goat Anti- Rabbit IgG (H+L)	Invitrogen	Cat#A-11011
Alexa Fluor 647 Goat Anti-Rat IgG (H+L)	Invitrogen	Cat#A-21247

**Chemicals, Peptides, and Recombinant Proteins**		

3-Isobutyl-1-methylxanthine (IBMX)	Sigma-Aldrich	Cat#I7018
AZD7762	Sigma-Aldrich	Cat#SML0350
PMS (pregnant mare’s serum)	Intervet Austria	Folligon 1000 I.E.
Caffeine	Sigma-Aldrich	Cat#C8960
Cytochalasin B	Sigma-Aldrich	Cat#C2743
Dimethyloxalylglycine (DMOG)	Sigma-Aldrich	Cat#D3695
Fetal Bovine Serum (FBS)	GIBCO	Cat#10270106
Fetuin	Sigma-Aldrich	Cat#F6131
Goat serum (normal)	Vector Labs	Cat#VECS-1000
hCG (human chorionic gonadotropin)	Intervet Austria	Chorulon 1500 I.E.
Hyaluronidase	Sigma-Aldrich	Cat#H3506
Hydroxyurea	Sigma-Aldrich	Cat#H8627
KU-55933	Sigma-Aldrich	Cat#SML1109
M16 media	Home-made	N/A
M2 media	Home-made	N/A
Mineral oil	Sigma-Aldrich	Cat#M8410
NSC109555	Sigma-Aldrich	Cat#SML0781
NU7026	Sigma-Aldrich	Cat#N1537
PBS, pH7.2 (Ca/Mg free)	Invitrogen	Cat#20012019
PF-477736	Sigma-Aldrich	Cat#PZ0186
Research Vitro Fert media	Cook Austria GmbH	Cat#K-RVFE-50
Research Vitro Wash media	Cook Austria GmbH	Cat#K-RVVWA-50
Tyrode’s solution, acidic	Sigma-Aldrich	Cat#T1788
VE-821	Sigma-Aldrich	Cat#SML1415
Vectashield with DAPI	Vector Labs	Cat#VECH-1200

**Critical Commercial Assays**		

Click-iT EdU Alexa Fluor 647 imaging kit	Invitrogen	Cat#C10340
mMessage mMachine T3 kit	Ambion	Cat#AM1348

**Experimental Models: Organisms/Strains**		

Mouse: B6/129SV	Tachibana-Konwalski, K., IMBA, Vienna, Austria, (Tachibana-Konwalski et al., 2010)	N/A
Mouse: B6/CBAF1	IMBA, Vienna, Austria	N/A
Mouse: *Tg(Zp3-Cre)*^*3Mrt/J*^	The Jackson Laboratory, (Lewandoski et al., 1997)	JAX:003394
Mouse: *Scc1*^*fl/fl*^	Tachibana-Konwalski, K., IMBA, Vienna, Austria, (Seitan et al., 2011)	N/A
Mouse: *Scc1*^*fl/fl*^*Tg(Zp3-Cre)*^*3Mrt/J*^	This paper	N/A
Mouse: *Xrcc1*^*tm1Pmc*^ (*Xrcc1*^*fl/fl*^*)*	McKinnon, P.J., Dpt. of Genetics, St Jude Children’s Research Hospital, Memphis, TN 38105, (Lee et al., 2009)	N/A
Mouse: *Xrcc1*^*tm1Pmc*^ (*Xrcc1*^*fl/fl*^*) Tg(Zp3-Cre)*^*3Mrt/J*^	This paper	N/A

**Software and Algorithms**		

GraphPad Prism	GraphPad Software Inc	http://www.graphpad.com/scientific-software/prism/
ImageJ	NIH	https://imagej.nih.gov/ij/

**Other**		

Grace Bio-Labs SecureSeal imaging spacer	Sigma-Aldrich	Cat#GBL654002
Thermo Scientific Nunc Lab-Tek Chambered Coverglass	Fisher Scientific	Cat#10778091

### Contact for Reagent and Resource Sharing

Further information and requests for reagents may be directed to Lead Contact Kikuë Tachibana-Konwalski (kikue.tachibana@imba.oeaw.ac.at).

### Experimental Model and Subject Details

#### Animals

Generation of *Xrcc1*^*fl/fl*^ and *Scc1*^*fl/fl*^ mice was described previously (Lee et al., 2009, Seitan et al., 2011). Experimental animals were female offspring obtained from breeding homozygous floxed females with homozygous floxed males positive for *Tg(Zp3-Cre)* (Lan et al., 2004, Lewandoski et al., 1997). These mice were bred on a mixed B6/129SV genetic background. B6/CBAF1 male mice were used for natural mating or in vitro fertilization. Mice were housed under a 14 hr light / 10 hr dark cycle in individually ventilated cages with continous access to food and water supply. Animal experiments were carried out in agreement with the authorizing committee of the Institute of Molecular Biotechnology of the Austrian Academy of Sciences (IMBA, Vienna, Austria) according to the Austrian Animal Welfare law and the international guiding principles for biomedical research involving animals (CIOMS, the Council for International Organizations of Medical Sciences).

### Method Details

#### Retrieval and In Vitro Culturing of Oocytes

Fully grown oocytes, naturally arrested in dictyate of prophase I, were isolated by physical disaggregation of ovaries from 2-5 months old females in M2 medium supplemented with 0.2 mM of the phosphodiesterase inhibitor 3-Isobutyl-1-methylxanthine (IBMX, Sigma-Aldrich) at 37°C. Mature oocytes were selected according to appearance (size, central nucleus, smooth zona pellucida) and cultured in M16 media supplemented with IBMX at 37°C and 5% CO_2_. Resumption of meiosis I was triggered by wash out of IBMX and successive culturing in M16 media. Timely nuclear envelope breakdown within 90 min post-release from IBMX was used as further indicator of oocyte maturity. Oocyte cultivation was performed in ∼40 μl drops covered with mineral oil (Sigma-Aldrich).

#### Retrieval and In Vitro Culturing of Zygotes

For retrieval of zygotes, timed mating was performed with 3-5 week old female mice superovulated by consecutive intraperitoneal injections of 5 U pregnant mare’s serum (PMS, Intervet Austria) followed by 5 U of human chorionic gonadotropin (hCG, Intervet Austria) 48 hr later. The second injection is used as reference time point for all experiments (time post-superovulation). Females were sacrificed 17-18 hr post-hCG. Zygotes were released from cumulus cells by brief incubation with 300 μg/ml hyaluronidase (Sigma-Aldrich). Cells were cultured in ∼40 μl drops of M16 media covered with mineral oil (Sigma-Aldrich) and incubated at 37°C and 5% CO_2_. Selection of zygotes for experiments was based on scoring for formation of visible pronuclei at 20 hr post-hCG.

#### Generation of Parthenogenotes

Female mice were superovulated as described above and sacrificed at 16 hr post-hCG injection. Metaphase II oocytes surrounded by cumulus cells were released into 7% ethanol in PBS pH 7.2 (Ca and Mg free; Invitrogen) and incubated for 5 min. Cell masses were washed through M16 media and incubated at 37°C and 5% CO_2_ in ∼40μl drops covered with mineral oil (Sigma-Aldrich). Media was supplemented with 5 μg/ml cytochalasin B (Sigma-Aldrich) for generation of diploid parthenogenotes. Cumulus cells were removed 5-6 hr later by brief incubation in 300 μg/ml hyaluronidase (Sigma-Aldrich). Activated oocytes were classified by formation of one or two visible pronuclei for haploid or diploid parthenogenotes, respectively. For treatment with Hydroxyurea (Sigma-Aldrich), parthenogenotes were incubated in 20 mM Hydroxyurea in M16 media after scoring for visible pronuclei.

#### In Vitro Maturation and Fertilization

Isolation of oocytes was performed as described above but media was supplemented with with Fetal Bovine Serum (FBS, GIBCO) and fetuin (Sigma-Aldrich). Incubation was done at 37°C and low oxygen (5% CO_2_, 5% O_2_, 90% N_2_). Mature oocytes were microinjected with mRNA as described below and subsequently released from IBMX to initiate in vitro maturation to metaphase II eggs. Next, in vitro fertilization was performed using sperm isolated from the *Cauda epididymidis* and *Vas deferens* of male B6/CBAF1 mice. Metaphase II eggs were briefly washed through Research Vitro Wash media (Cook Austria GmbH) before incubation with capacitated sperm in Research Vitro Fert media (Cook Austria GmbH). Formation of visible pronuclei that indicates zygotes was scored at 7-8 hr post-fertilization.

#### Microinjection

Microinjection of in vitro transcribed mRNA soluted in RNase-free water (mMessage mMachine T3 kit, Ambion) was performed in M2 media using a Pneumatic PicoPump (World Precision Instruments) and hydraulic micromanipulator (Narishige) mounted onto a Zeiss Axiovert 200 microscope equipped with a 10x/0.3 EC plan-neofluar and 40x/0.6 LD Apochromat objective. Following mRNA concentrations have been injected (alphabetical order): 0.7 pmol CenpB-EGFP; 2.3 pmol hGeminin^L26A^; 0.5 pmol H2B-mCherry; 2.3 pmol Mad2-flag; 0.4 pmol Scc1 (wild-type and mutants); 2 pmol Securin-EGFP; 2.3 pmol TEV protease.

#### Time-Lapse Microscopy

Time-lapse microscopy was performed using a customized Zeiss LSM510 META confocal microscope together with an adapted EMBL-developed tracking macro (Rabut and Ellenberg, 2004). Cells were mounted to the microscope using Thermo Scientific Nunc Lab-Tek Chambered Coverglass (Fisher Scientific) and incubated in M16 media covered with mineral oil (Sigma-Aldrich) at at 37°C and 5% CO_2_. Kinetic analysis was performed using a plan-apochromat 25x/0.8 oil immersion objective and image acquisition every 15 min. For high-resolution images of individual cells a P C-Apochromat 63x/1.2 water immersion objective lens was used.

#### Drug Treatment of Zygotes

For kinase inhibitor experiments zygotes were incubated after scoring for visible pronuclei at 20 hr post-hCG (corresponding to early/mid G1 phase) in continuous presence of the respective inhibitor [10 μM KU-55933 (Hickson et al., 2004), Sigma-Aldrich; 10 μM VE-821 (Charrier et al., 2011, Reaper et al., 2011), Sigma-Aldrich; 10 μM NU7026 (Veuger et al., 2003), Sigma-Aldrich; 2 mM caffeine (Sarkaria et al., 1999), Sigma-Aldrich; 100 nM AZD7762 (Zabludoff et al., 2008), Sigma-Aldrich; 5 μM NSC109555 (Jobson et al., 2007), Sigma-Aldrich; 10 nM PF-477736 (Blasina et al., 2008), Sigma-Aldrich]. Incubation in Tet3 inhibitor [1 mM Dimethyloxalylglycine (DMOG) (Amouroux et al., 2016), Sigma-Aldrich] directly followed zygote isolation at 17-18h post-hCG.

#### In Situ Fixation and Immunofluorescence

To allow proper cell-cycle phase staging in relation to DNA replication, zygotes were pulsed with 1 mM EdU or BrdU (Invitrogen) before fixation. As positive control a small fraction of zygotes were fixed during S phase after continuous incubation in presence of EdU or BrdU. In situ fixation of oocytes or zygotes was performed by removal of the zona pellucida using acidic Tyrode’s solution (Sigma-Aldrich) followed by fixation in 4% PFA in 0.5 x PBS with 25 mM HEPES. Cells pulsed with EdU were processed according to the manual of the Click-iT^**®**^ EdU Alexa Fluor 647 imaging kit (Invitrogen). Intermediate washes were done in 0.2% Triton X-100 in PBS (PBTX). Blocking was performed using 10% goat serum (Vector Labs) in PBTX. Following primary antibodies were used: anti-Scc1 (1:500; Millipore, #05-908), anti-γH2AX (1:500; Abcam, #ab22551), anti-Rad51 (1:500; Santa Cruz, #sc-8349), anti-5mC (1:500; Eurogentec, #BI-MECY-0100), anti-5hmC (1:500; Active Motif, #39769), anti-BrdU (1:50; Abcam, #ab6326), anti-Xrcc1 (1:300; Serotec, # AHP832). Alexa Fluor^**®**^ 488, 568 or 647 secondary antibodies (Invitrogen) were used to detect primary antibodies. Cells were mounted in Vectashield^**®**^ plus DAPI (Vector Labs) using Grace Bio-Labs SecureSeal imaging spacer (Sigma-Aldrich) to preserve 3D integrity.

Note, detection of Rad51 or chromatin bound Xrcc1 required pre-extraction before fixation and was performed with minor modifications as described in (Hajkova et al., 2010). Briefly, zona pellucida has been left intact and cells were incubated in ice-cold extraction buffer (50 mM NaCl; 3 mM MgCl_2_; 300 mM Sucrose; 25 mM HEPES; 0.5% Triton X-100) for 10 min on ice. Cells were washed briefly through ice-cold extraction buffer without Triton X-100. Fixation and immunofluorescence staining was performed as described above. Cells were washed through increasing Vectashield concentrations before final mounting to avoid the zona pellucida to collapse.

Note, detection of 5mC, 5hmC and BrdU required DNA denaturation with 4 M HCl for 10 min after fixation. Neutralization was performed in 0.1 M Tris pH 8 in PBS. Cells were post-fixed and immunofluorescence detection was performed as described above.

Image acquisition was performed on a Zeiss LSM780 confocal microscope equipped with plan-apochromat 63x/1.4 NA oil immersion objective.

### Quantification and Statistical Analysis

#### Image Analysis

Image analysis was performed using ImageJ software (NIH). Mean intensity was measured within a defined nuclear area of each oocyte or zygote. Securin-EGFP intensity curves were generated with the MultiThresholder and Time Series Analyzer Plugin. Securin degradation curves for oocytes were normalized to the Securin-EGFP intensity at the time of meiotic entry (nuclear envelope breakdown). Foci analysis of γH2AX and Rad51 was performed by analysis of particles > 0.5 μm^2^ corresponding to the foci size covered by the applied z stack range. Thresholds were kept constant within each experiment. Measurement of the metaphase plate height was performed on the 3D projection of the time point before anaphase to allow measurement perpendicular to the metaphase plate.

#### Statistics

Statistical parameters and tests are reported in the Figures and corresponding Figure Legends. Statistical analysis was done using GraphPad Prism version 6.0 (GraphPad Software Inc). D’Agostino-Pearson omnibus normality test was performed to test for Gaussian data distribution. Parametric unpaired t test was performed for datasets following Gaussian distribution, while the nonparametric unpaired Mann-Whitney test was used for datasets not passing the normality test. Data collection and analysis were not performed blind to the conditions of the experiments.

## Author Contributions

Conceptualization, Methodology, and Writing, S.L. and K.T.-K.; Validation, Formal Analysis, Investigation, and Visualization, S.L.; Supervision, Project Administration, and Funding Acquisition, K.T.-K.
