# Supplementary material for: A Surveillance Mechanism Ensures Repair of DNA Lesions during Zygotic Reprogramming
Source: Cell. 2016 Dec 15;167(7):1774–1787.e13. doi: 10.1016/j.cell.2016.11.009 (PMC5161750; doi:10.1016/j.cell.2016.11.009)
Supplement: Document S1. Tables S1 and S2 [file mmc1.pdf]

**Cell, Volume *167***

## Supplemental Information

# A Surveillance Mechanism Ensures Repair of DNA Lesions during Zygotic Reprogramming

**Sabrina Ladstätter and Kikuë Tachibana-Konwalski**

**Supplemental Table S1, Related to Figure 2, Figure S3**

**Numbers of mature oocytes isolated from *$Scc1^{fl/fl}$ and $Scc1^{fl/fl}$ (Tg)Zp3-Cre* (*$Scc1^{\Delta/\Delta}$*) female mice.** Mature oocytes are defined by appearance (size, central nucleus, smooth zona pellucida) and timely extrusion of the 1st polar body around 10 h post-release from phosphodiesterase inhibitor IBMX.

| | ***$Scc1^{fl/fl}$*** | | | ***$Scc1^{\Delta/\Delta}$*** | | |
|---|---|---|---|---|---|---|
| | Number of oocytes | Number of females | Oocytes per female | Number of oocytes | Number of females | Oocytes per female |
| Experiment 1 | 24 | 3 | 8 | 23 | 3 | 8 |
| Experiment 2 | 31 | 4 | 8 | 45 | 5 | 9 |
| Experiment 3 | 21 | 4 | 5 | 26 | 3 | 9 |
| Experiment 4 | 21 | 2 | 11 | 47 | 6 | 8 |
| Experiment 5 | 17 | 2 | 9 | 41 | 5 | 8 |
| Experiment 6 | 37 | 3 | 12 | 56 | 6 | 9 |
| Experiment 7 | 24 | 3 | 8 | 29 | 3 | 10 |
| Experiment 8 | 33 | 3 | 11 | 43 | 4 | 11 |
| **Total** | **208** | **24** | **9 ± 2** | **310** | **35** | **9 ± 1** |

**Supplemental Table S2, Related to Figure 2**

**Numbers of zygotes obtained after natural mating of superovulated female mice.** Zygotes are isolated 17-18 h post-superovulation and scored for formation of visible pronuclei at 20 h post-superovulation.

| | ***Scc1**$^{fl}$* | | | ***Scc1**$^{\Delta}$* | | |
|---|---|---|---|---|---|---|
| | Number of zygotes | Number of females | Zygotes per female | Number of zygotes | Number of females | Zygotes per female |
| Experiment 1 | 48 | 4 | 12 | 34 | 3 | 11 |
| Experiment 2 | 67 | 8 | 8 | 47 | 5 | 9 |
| Experiment 3 | 15 | 2 | 8 | 44 | 4 | 11 |
| Experiment 4 | 25 | 4 | 6 | 49 | 5 | 10 |
| Experiment 5 | 45 | 6 | 8 | 47 | 6 | 8 |
| Experiment 6 | 10 | 1 | 10 | 15 | 2 | 8 |
| Experiment 7 | 33 | 5 | 7 | 37 | 5 | 7 |
| Experiment 8 | 19 | 3 | 6 | 38 | 5 | 8 |
| **Total** | **262** | **33** | **8 ± 2** | **311** | **35** | **9 ± 2** |
